# COVID-box Experiences of Patients and Health Care Professionals (COVID-box Project): Single-Center, Retrospective, Observational Study

**DOI:** 10.2196/38263

**Published:** 2022-07-28

**Authors:** Ebru Dirikgil, Kim Brons, Michael Duindam, Geert H Groeneveld, J J Miranda Geelhoed, Christian Heringhaus, Paul J M van der Boog, Ton J Rabelink, Willem Jan W Bos, Niels H Chavannes, Douwe E Atsma, Y K Onno Teng

**Affiliations:** 1 Department of Nephrology Leiden University Medical Center Leiden Netherlands; 2 Directorate of Quality and Patient Safety Leiden University Medical Center Leiden Netherlands; 3 Department of Infectious diseases Leiden University Medical Center Leiden Netherlands; 4 Department of Pulmonology Leiden University Medical Center Leiden Netherlands; 5 Department of Emergency Medicine Leiden University Medical Center Leiden Netherlands; 6 Department of Internal Medicine St Antonius Hospital Nieuwegein Netherlands; 7 Department of Public Health and Primary Care Leiden University Medical Center Leiden Netherlands; 8 National eHealth Living Lab Leiden University Medical Center Leiden Netherlands; 9 Department of Cardiology Leiden University Medical Center Leiden Netherlands

**Keywords:** COVID-19, digital health, eHealth, telemedicine, telemonitoring, hospital admission, health care professional, thematic analysis, user satisfaction, usability, home monitoring, health care system

## Abstract

**Background:**

During the COVID-19 pandemic, several home monitoring programs have described the success of reducing hospital admissions, but only a few studies have investigated the experiences of patients and health care professionals.

**Objective:**

The objective of our study was to determine patients’ and health care professionals’ experiences and satisfaction with employing the COVID-box.

**Methods:**

In this single-center, retrospective, observational study, patients and health care professionals were asked to anonymously fill out multiple-choice questionnaires with questions on a 5-point or 10-point Likert scale. The themes addressed by patients were the sense of reassurance and safety, experiences with teleconsultations, their appreciation for staying at home, and the instructions for using the COVID-box. The themes addressed by health care professionals who treated patients with the COVID-box were the characteristics of the COVID-box, the technical support service and general satisfaction, and their expectations and support for this telemonitoring concept. Scores were interpreted as *insufficient* (≤2 or ≤5, respectively), *sufficient* (3 or 6-7, respectively), or *good* (≥4 or ≥8, respectively) on a 5-point or 10-point Likert scale.

**Results:**

A total of 117 patients and 25 health care professionals filled out the questionnaires. The median score was 4 (IQR 4-5) for the sense of safety, the appreciation for staying at home, and experiences with teleconsultations, with good scores from 76.5% (88/115), 86% (56/65), and 83.6% (92/110) of the patients, respectively. Further, 74.4% (87/117) of the patients scored the home monitoring program with a score of ≥8. Health care professionals scored the COVID-box with a minimum median score of 7 (IQR 7-10) on a 10-point scale for all domains (ie, the characteristics of the COVID-box and the technical support service and general satisfaction). For the sense of safety, user-friendliness, and additional value of the COVID-box, the median scores were 8 (IQR 8-10), 8 (IQR 7-9), and 10 (IQR 8-10), respectively, with good scores from 86% (19/22), 75% (15/20), and 96% (24/25) of the health care professionals, respectively. All health care professionals (25/25, 100%) gave a score of ≥8 for supporting this home monitoring concept, with a median score of 10 (IQR 10-10).

**Conclusions:**

The positive experiences and satisfaction of involved users are key factors for the successful implementation of a novel eHealth solution. In our study, patients, as well as health care professionals, were highly satisfied with the use of the home monitoring program—the COVID-box project. Remote home monitoring may be an effective approach in cases of increased demand for hospital care and high pressure on health care systems.

## Introduction

The COVID-19 pandemic resulted in an increased demand for hospital care. To keep up with this surging demand, home monitoring was implemented in many countries to avoid unnecessary hospital admissions and detect clinical deterioration in patients at an earlier stage to allow for timely admission and readmission [1].

At the Leiden University Medical Center (LUMC), the Netherlands, we developed the COVID-box project, which is a home monitoring program for patients with (suspected) COVID-19. After a hospital or emergency department visit, patients with (suspected) COVID-19 receive Bluetooth-connected devices (blood pressure monitor, pulse oximeter, and thermometer) and instructions for monitoring their vital parameters 3 times per day, combined with daily teleconsultations carried out by a health care professional. Once the patients get home, the COVID-box team calls the patients to help with the installation of devices and answer questions. The COVID-box team is reachable during office hours for solving logistics issues and answering questions from patients and health care professionals. A detailed description of the telemonitoring program was published previously [2].

The implementation of several home monitoring programs has resulted in a reduction in hospital admissions by allowing for the safe survey of clinical symptoms and vitals [3-9]. Although many observational studies have studied the effectiveness of home monitoring, few studies have reported on patients’ and doctors’ experiences with telemonitoring [5,10-13]. We focused on the COVID-box experiences of patients and health care professionals.

## Methods

### Ethics Approval

Central ethical approval was obtained for this study from the medical ethics committee of the LUMC.

### Study Design

This retrospective observational study was conducted as part of the COVID-box project, which was initiated at the LUMC in May 2020 upon the first wave of the pandemic. A detailed operational description of the COVID-box project was previously reported [2]. In this study, we evaluated patients with COVID-19 and their experiences with telemonitoring by surveying patients after the completion of the telemonitoring phase and full recovery. Patients were asked to anonymously fill out questionnaires regarding the sense of reassurance and safety, experiences with teleconsultations, their appreciation for staying at home, and the instructions for the COVID-box. Health care professionals who treated patients with the COVID-box were given separate questionnaires regarding the characteristics of the COVID-box, the technical support service and general satisfaction (estimated patient satisfaction), and their expectations and support for this telemonitoring concept. All questions were multiple-choice questions on a 5-point or 10-point Likert scale. For this study, scores were interpreted as follows: on a 5-point Likert scale, scores of ≤2 were *insufficient* scores, scores of 3 were *sufficient* scores, and scores of ≥5 were *good* scores; on a 10-point scale, scores of ≤5 were marked as *insufficient*, scores of 6 to 7 were marked as *sufficient*, and scores of ≥8 were marked as *good*. Unvalidated questionnaires were developed by members of the Department of the Directorate of Quality and Patient Safety and clinicians from the Department of Internal Medicine. The questionnaires were web-based and anonymous.

### Statistics

Descriptive statistics were used to summarize the results. The scores given by patients and health care professionals are presented as medians with IQRs, and the number of patients and health care professionals are presented as absolute numbers and percentages. We used IBM SPSS Statistics Version 25 (IBM Corporation).

## Results

### Patients

**Figure 1:**
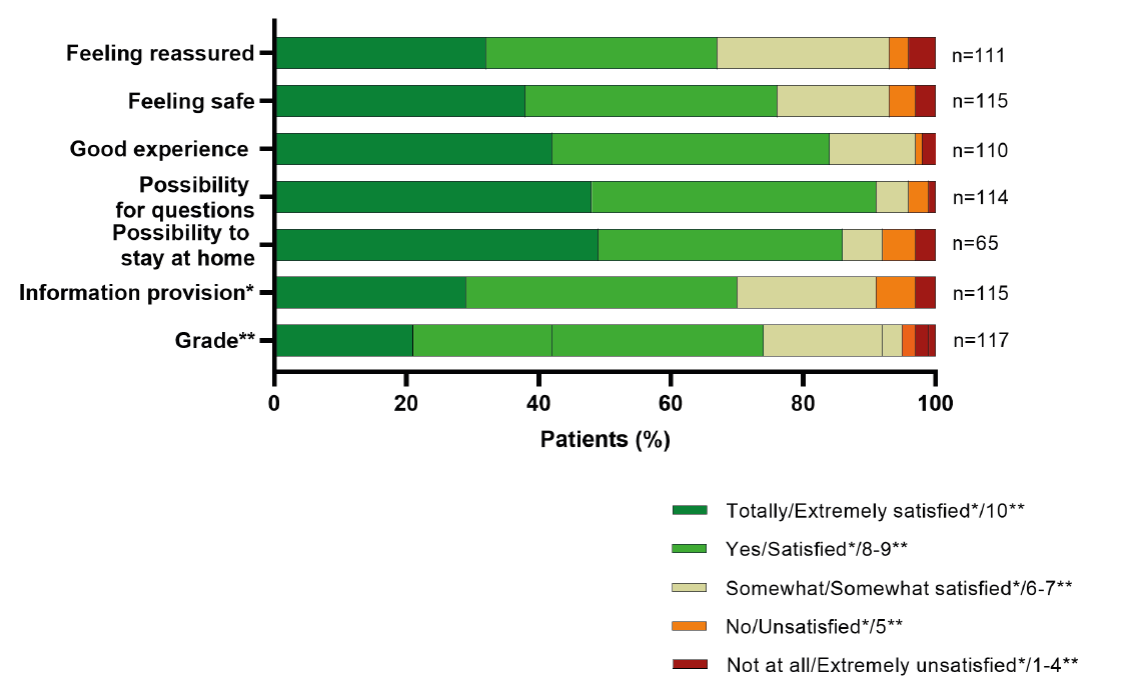
Patients' valuation. *"Information provision" ratings; **"Grade" ratings.

Of the first 300 patients who were monitored in the COVID-box project from June 2020 to March 2021, a total of 117 (39%) responded to a web-based survey (Multimedia Appendix 1). All of these patients underwent actual telemonitoring with at least 1 contact with a health care professional by using the COVID-box as a home monitoring tool. The results are summarized in Figure 1.

The median score for the sense of reassurance and for the instructions of the COVID-box was 4 (IQR 3-5), with good scores from 67.6% (75/111) and 70% (80/115) of patients, respectively. For the sense of safety, experiences with teleconsultations, the appreciation for staying at home, and the ability to ask questions about the disease and disease course, the median score was 4 (IQR 4-5); good scores were given by 76.5% (88/115), 83.6% (92/110), 86% (56/65), and 91.2% (104/114) of patients, respectively. Overall, 74.4% (87/117) of patients scored the home monitoring program with the COVID-box as *good*, with a score of 8 or higher.

### Health Care Professionals

#### Overview

**Figure 2:**
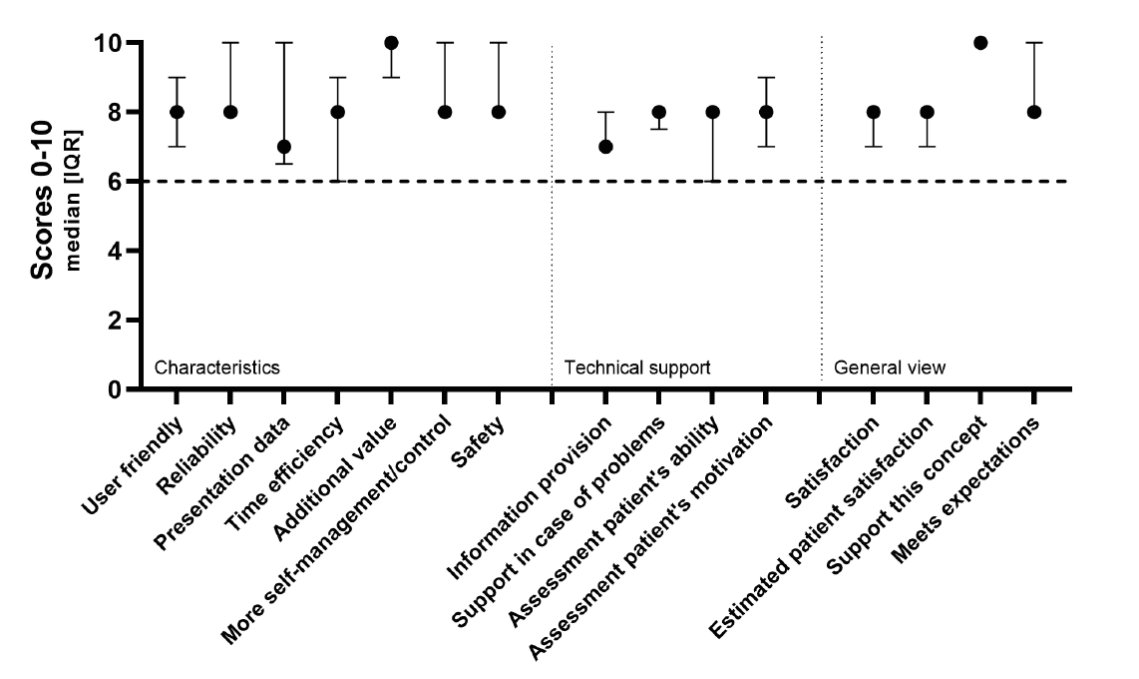
Health care professionals' valuation.

Of the 60 health care professionals approached, 25 (42%) filled out the questionnaire (Multimedia Appendix 2). Of these, 6 (24%) were specialists who worked at the emergency department, 5 (20%) were specialists from the Department of Internal Medicine (20%), 12 (48%) were residents, and 2 (8%) were nurse practitioners. The results are summarized in Figure 2.

#### Characteristics of the COVID-box

The median score for the sense of safety, reliability, and more self-management/control in patients was 8 (IQR 8-10), with good scores from 86% (19/22), 89% (8/9), and 92% (22/24) of health care professionals, respectively. For user-friendliness, data presentation, and time efficiency, the median scores were 8 (IQR 7-9), 7 (IQR 7-10), and 8 (IQR 6-9), respectively; good scores were given by 75% (15/20), 44% (4/9), and 55% (6/11) of health care professionals, respectively. The additional value of the COVID-box had a median score of 10 (IQR 8-10), with good scores from 96% (24/25) of health care professionals.

#### Technical Support Service (COVID-box Team)

For information provision and technical support for problems, the median scores were 7 (IQR 7-8) and 8 (IQR 7.5-8), respectively, with good scores from 37% (8/22) and 76% (13/17) of health care professionals, respectively. The median scores for the assessment of a patient’s ability and motivation were 8 (IQR 6-8) and 8 (IQR 7-9), respectively. A total of 57% (13/23) and 76% (16/21) of health care professionals, respectively, scored these items as *good*.

#### General Satisfaction

The general satisfaction of health care professionals and estimated patient satisfaction had a median score of 8 (IQR 7-8), and 60% (15/25) and 95% (18/19) of health care professionals scored these items as *good*, respectively. With regard to meeting the expectations for this home monitoring concept, good scores were given by 92% (22/24) of health care professionals, with a median score of 8 (IQR 8-10). With regard to supporting this home monitoring concept, the median score was 10 (IQR 10-10), with good scores from all health care professionals (25/25, 100%).

## Discussion

### Principal Findings

Our study demonstrates that the home monitoring of patients with COVID-19 is well appreciated by patients as well as health care professionals. Previous observational studies have shown the safety of the remote telemonitoring of patients with (suspected) COVID-19 and its efficacy in reducing hospitalization. Few studies have addressed user experience and patients’ and health care professionals’ satisfaction with telemonitoring. It is well established that the successful implementation of novel eHealth solutions is critically dependent on the positive experiences and satisfaction of involved users.

### Comparison With Prior Work

During the extraordinary situation of the COVID-19 pandemic, remote telemonitoring has been quickly implemented in different ways [5-7,10-13]. In general, many patients and health care professionals are very positive about this concept. Remote telemonitoring is used for conducting disease triage; reducing hospital admissions; and providing reassurance, disease and disease course information, and psychological support to clinically stable patients at home. Several studies have reported that patients appreciate all forms of remote telemonitoring (eg, the measuring of vital parameters, symptom recording, and daily teleconsultations) in various settings (eg, in primary care and after an emergency department visit or hospital admission) [5-7,10-13]. The important aspects are easy access to the program, good information provision, and the good quality of the service for the onboarding process. Importantly, older age does not seem to be a problem, as different studies have successfully included patients aged >50 years [5,7,11]. Patients have pointed out that video consultations are also highly appreciated. Self-evidently, the adherence of patients to telemonitoring is critical to its success. Nonadherence to telemonitoring among patients with COVID-19 has been reported when they feel too sick, forget to measure vital parameters, feel insufficiently informed, or experience quick improvements in disease symptoms [13]. Health care professionals are largely convinced of the benefits of remote telemonitoring, as long as a program is easy to use and it is possible to receive patient data correctly.

### Limitations

Given the observational and retrospective nature of our study, which was conducted during the COVID-19 pandemic, our study has several limitations that are noteworthy. First, the patient-reported experience measure questionnaires on telemonitoring a new disease, such as COVID-19, were not validated, and a formal validation of these questionnaires was out of the scope of this study. Second, the questionnaires were completed anonymously; therefore, internal consistency could not be reliably assessed. Lastly, the relatively low response rates (patients: 117/300, 39%; health care professionals: 25/60, 42%) could have introduced unwanted bias to the results of this study.

### Conclusion

In conclusion, the home monitoring of patients with COVID-19 is well appreciated by patients as well as health care professionals. This study demonstrated that patients felt safe and reassured with the home monitoring and daily teleconsultations in the COVID-box project. Additionally, health care professionals were satisfied with the safety and user-friendliness of the COVID-box. The acceptation of the COVID-box is critical for the successful implementation and expansion of home monitoring for patients with COVID-19 to relieve the burden on health care systems. Our findings could be especially relevant to the current perspectives on oral antiviral agents for the out-of-hospital treatment of patients with COVID-19.

## Appendix

Multimedia Appendix 1 Questionnaire for patients.

Multimedia Appendix 2 Questionnaire for healthcare professionals.

## Abbreviations

LUMC: Leiden University Medical Center
