# Supplementary material for: COVID-box Experiences of Patients and Health Care Professionals (COVID-box Project): Single-Center, Retrospective, Observational Study
Source: JMIR Form Res. 2022 Jul 28;6(7):e38263. doi: 10.2196/38263 (PMC9337621; doi:10.2196/38263)
Supplement: Multimedia Appendix 1 [file formative_v6i7e38263_app1.docx]

**Appendix 1. Questionnaire for patients**

In recent months you have participated in the "COVID-box program" in which you measured your vital parameters daily and had daily contact with one of our healthcare professionals.

We would like to ask about your experience with the COVID-box on the basis of a questionnaire. This questionnaire consists of 7 statements or questions.

**1. Did you feel reassured when you were allowed to go home with the COVID-box?**

not at all (1) | no (2) | somewhat (3) | yes (4) | totally (5)

**2. Did you feel safe at home when you were allowed to go home with the COVID-box?**

not at all (1) | no (2) | somewhat (3) | yes (4) | totally (5)

**3. Did you have a good experience with the COVID-box and the daily communication?**

not at all (1) | no (2) | somewhat (3) | yes (4) | totally (5)

**4. Did you have the opportunity to ask questions to the healthcare professional you spoke to on a daily basis?**

not at all (1) | no (2) | somewhat (3) | yes (4) | totally (5)

**5. Did you like the possibility to stay at home and not being hospitalized?**

not at all (1) | no (2) | somewhat (3) | yes (4) | totally (5)

**6. How satisfied or unsatisfied were you with the information provided by the COVID-box team when you started home monitoring?**

extremely unsatisfied (1) | unsatisfied (2) | somewhat satisfied (3) | satisfied (4) | extremely satisfied (5)

**7. What grade would you give the COVID box program?**

1 | 2 | 3 | 4 | 5 | 6 | 7 | 8 | 9 | 10

*Additional information:*

*Question 1, 2, 3, 4, 5 and 6 were on a 5-point Likert scale. Question 7 was on a 10-point scale.*

| *5-point Likert scale* | *10-point scale* |
| --- | --- |
| *1-2: insufficient*  *3: sufficient*  *4-5: good* | *1-5: insufficient*  *6-7: sufficient*  *8-10: good* |
