# Supplementary material for: COVID-box Experiences of Patients and Health Care Professionals (COVID-box Project): Single-Center, Retrospective, Observational Study
Source: JMIR Form Res. 2022 Jul 28;6(7):e38263. doi: 10.2196/38263 (PMC9337621; doi:10.2196/38263)
Supplement: Multimedia Appendix 2 [file formative_v6i7e38263_app2.docx]

**Appendix 2. Questionnaire for healthcare professionals**

Because of the severe pressure on national healthcare systems caused by the COVID-19 pandemic, we developed the “COVID-box program”, in which patients measure their vital parameters three times per day and have daily consultations with one healthcare professional. With this program, we hope that it is possible to avoid unnecessary hospital admissions and to detect clinical deterioration at an earlier stage for timely (re)admission.

In the past few months, you have used the COVID-box regularly and we would like to evaluate your experience with the COVID-box.

We would like to ask you 15 questions, divided in 3 categories. You could answer on a scale of 1 to 10.

**COVID-box characteristics**

**1. How satisfied or unsatisfied are you with the user-friendliness of the COVID-box?**

1 | 2 | 3 | 4 | 5 | 6 | 7 | 8 | 9 | 10

**2. How satisfied or unsatisfied are you with the reliability of the COVID-box?**

1 | 2 | 3 | 4 | 5 | 6 | 7 | 8 | 9 | 10

**3. How satisfied or unsatisfied are you with the measurement data presented in the electronic patient record?**

1 | 2 | 3 | 4 | 5 | 6 | 7 | 8 | 9 | 10

**4. How satisfied or unsatisfied are you with the time efficiency when using the COVID-box?**

1 | 2 | 3 | 4 | 5 | 6 | 7 | 8 | 9 | 10

**5. How satisfied or unsatisfied are you with the added value of the COVID-box?**

1 | 2 | 3 | 4 | 5 | 6 | 7 | 8 | 9 | 10

**6. How satisfied or unsatisfied are you with the self-management that the COVID-box offers patients?**

1 | 2 | 3 | 4 | 5 | 6 | 7 | 8 | 9 | 10

**7. How safe do you feel when a patient goes home with the COVID-box (on a scale of 1-10)?**

1 | 2 | 3 | 4 | 5 | 6 | 7 | 8 | 9 | 10

**COVID-box support team**

**8. How satisfied or unsatisfied are you with the information provided by the COVID-box team when you start home monitoring?**

1 | 2 | 3 | 4 | 5 | 6 | 7 | 8 | 9 | 10

**9. How satisfied or unsatisfied are you with the support when technical problems arise?**

1 | 2 | 3 | 4 | 5 | 6 | 7 | 8 | 9 | 10

**10. How satisfied or unsatisfied are you with the correctness of the assessment of whether patients are able to use the COVID box?**

1 | 2 | 3 | 4 | 5 | 6 | 7 | 8 | 9 | 10

**11. How satisfied or unsatisfied are you with the correctness of the assessment of whether patients have sufficient motivation to use the COVID box?**

1 | 2 | 3 | 4 | 5 | 6 | 7 | 8 | 9 | 10

**General**

**12. How satisfied or dissatisfied are you with the COVID box in general?**

1 | 2 | 3 | 4 | 5 | 6 | 7 | 8 | 9 | 10

**13. How satisfied or dissatisfied do you think patients are with the COVID box in general?**

1 | 2 | 3 | 4 | 5 | 6 | 7 | 8 | 9 | 10

**14. Do you support the use of the COVID box (on a scale of 1-10)?**

1 | 2 | 3 | 4 | 5 | 6 | 7 | 8 | 9 | 10

**15. To what extent does the COVIX box meet your expectations (on a scale of 1-10)?**

1 | 2 | 3 | 4 | 5 | 6 | 7 | 8 | 9 | 10

**16. Should we continue this COVID-box program?**no | yes | I do not know

**17. What aspect could be improved?**

[Free text]

*Additional information:*

*All questions were on a 10-point scale.*

| *10-point scale* |
| --- |
| *1-5: insufficient*  *6-7: sufficient*  *8-10: good* |
